# Establishment of open-source semi-automated behavioral analysis system and quantification of the difference of sexual motivation between laboratory and wild strains

**DOI:** 10.1038/s41598-021-90225-3

**Published:** 2021-05-25

**Authors:** Soma Tomihara, Yoshitaka Oka, Shinji Kanda

**Affiliations:** 1 Department of Biological Sciences, Graduate School of Science, The University of Tokyo, 7-3-1 Hongo, Bunkyo, Tokyo 113-0033 Japan; 2 Laboratory of Physiology, Atmosphere and Ocean Research Institute, The University of Tokyo, 5-1-5 Kashiwanoha, Kashiwa, Chiba 277-8564 Japan

**Keywords:** Behavioural methods, Animal behaviour

## Abstract

Behavioral analysis plays an important role in wide variety of biological studies, but behavioral recordings often tend to be laborious and are associated with inevitable human-errors. It also takes much time to perform manual behavioral analyses while replaying the videos. On the other hand, presently available automated recording/analysis systems are often specialized for certain types of behavior of specific animals. Here, we established an open-source behavioral recording system using Raspberry Pi, which automatically performs video-recording and systematic file-sorting, and the behavioral recording can be performed more efficiently, without unintentional human operational errors. We also developed an Excel macro that enables us to easily perform behavioral annotation with simple manipulation. Thus, we succeeded in developing an analysis suite that mitigates human tasks and thus reduces human errors. By using this suite, we analyzed the sexual behavior of a laboratory and a wild medaka strain and found a difference in sexual motivation presumably resulting from domestication.

## Introduction

Animal behavior is one of the most important factors that represent the animal’s features, and the behavioral analysis often gives a lot of information complementally to the other analyses using methods in physiology, in vitro, etc. In addition to the behavioral analyses of wildtype animals, the recent development of genetic tools such as genome editing technology^1–4^ has increased the importance of analyzing behaviors of genetically modified animals as the most salient phenotypes. Thus, the demands of behavioral analyses are increasing, and the easier and more efficient method has been awaited.

So far, the majority of researchers have recorded the videos of animal behavior by using video camera and manually annotated the behavioral events while replaying the videos^5–11^. However, simultaneous video-recording inevitably tends to be associated with human-errors with increasing number of cameras. After video recording, it is also a confusing task to transfer the data from multiple video cameras to a storage, change their filenames systematically, and sometimes encode them. Furthermore, it is also painstaking to replay the entire video, pause and identify each behavioral event, and manually record the timing and/or duration of each behavioral event. Thus, the manual behavioral annotation takes much time. While there are some automated recording/analysis systems for the solution of these problems^12–21^, these are exclusively designed for certain species and/or behavior, and such systems often may not be applicable to the studies of non-model animals or rare behaviors. In addition, although commercially available systems are convenient and sophisticated, they are often expensive. Thus, such conventional onerous methods have been the only choice to analyze behaviors for many researchers.

In the present study, we established an open-source automated behavioral recording/file-sorting system with a single-board computer, Raspberry Pi™ and performed behavioral analysis efficiently without unintentional human errors caused by experimental operations. Raspberry Pi is an inexpensive credit card-sized computer, and we can remotely operate multiple Raspberry Pi at the same time via wireless local area network (WLAN) connections. By utilizing them, we have succeeded in automating a large part of experimental procedure of behavioral analysis: video-recording by multiple cameras, file-naming, encoding, and transferring video data from multiple cameras to a network-attached storage (NAS). In addition, Raspberry Pi can switch on and off external devices via general-purpose input/output (GPIO) pins and a simple relay circuit, which enables to automate simple experimental manipulations, such as feeding and illumination. This customization reduced the experimental procedures and made efficient/reproducible analyses of behaviors at low cost.

Furthermore, we established a Microsoft Excel macro for behavioral annotation. By using this tool, it became possible to annotate the behavioral events by only pressing the assigned PC keys when the behaviors of interest occur. As this tool makes manual notetaking unnecessary, the time taken for quantification of the behaviors was drastically shortened. This tool also enables the generation of a raster plot that indicates the time course of the behaviors, thereby making it easier to get an overview of the behavioral transition.

Using the combination of these two, the behavioral analyses can be carried out more efficiently, and consequently it may mitigate human errors. In the present study, we analyzed and compared the sexual behaviors of a laboratory and a wild strain of teleost medaka, and the time spent for the analysis was drastically shortened compared to the conventional method. Here, we found a difference in the sexual motivation between the strains probably caused by the different selection pressure in the laboratory and the wild environments.

## Materials and methods

### Hardware

**Figure 1:**
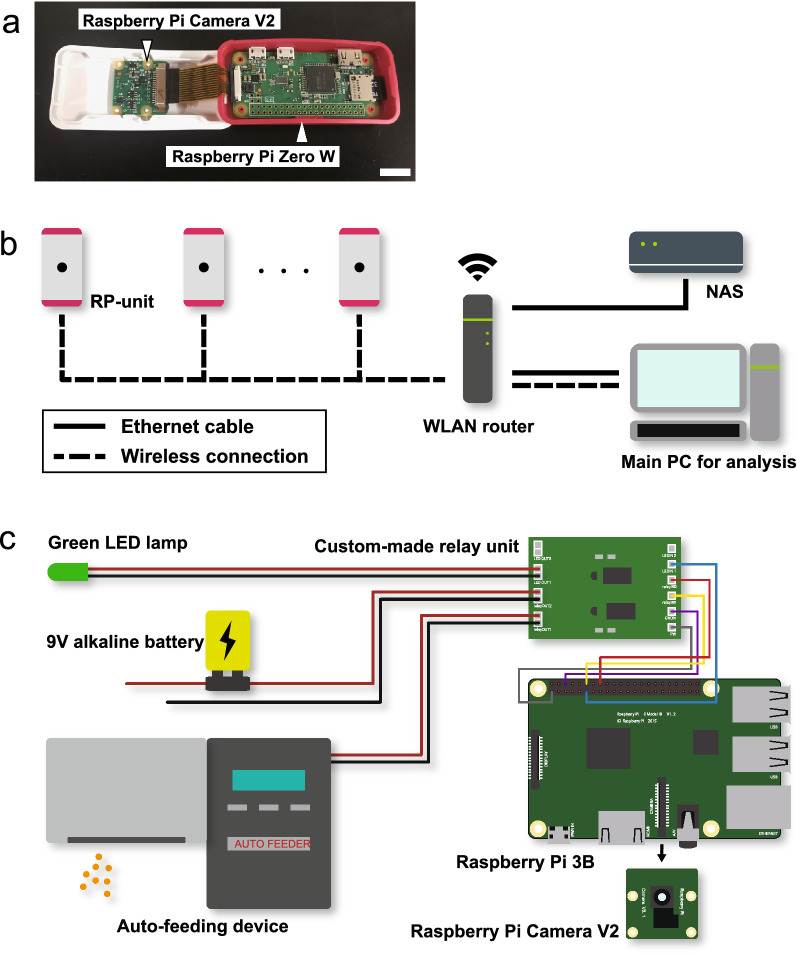
Hardware setup. (**a**) Photograph of RP-unit with their cases open. Raspberry Pi Camera V2 is connected to Raspberry Pi Zero W through the flat flexible cable. Scale bar indicates 1 cm. (**b**) Connection diagram of automated behavioral recording system. 16 RP-units are wirelessly connected to WLAN, while NAS is connected to WLAN router through Ethernet cable. We accessed and operated RP-units through SSH remote login by using a Windows PC, which is represented as “Main PC for analysis” on this figure. (**c**) Schematic diagram of another unit for automated switching of LED lamp, auto-feeding device, and the electric circuit including 9 V alkaline battery. These external devices are connected a custom-made relay unit that is connected to GPIO pins on Raspberry Pi 3B. Raspberry Pi Camera V2 is also connected to Raspberry Pi 3B for video-recording. Raspberry Pi 3B can be replaced to Raspberry Pi Zero W.

Two versions of Raspberry Pi, Raspberry Pi Zero W (Raspberry Pi Foundation, Cambridge, UK) and Raspberry Pi 3B (Raspberry Pi Foundation) installed with Raspbian (ver.3.1.1; latest version of which is called Raspberry Pi OS) were used in the present study. Each component of video-recording (hereafter “RP-unit”) consists of a Raspberry Pi Zero W and a Raspberry Pi Camera V2 (Raspberry Pi Foundation), which is a camera module designated for Raspberry Pi (Fig. 1a). We built in each of them in the designated case for Raspberry Pi Zero W, Raspberry Pi Zero Case (Raspberry Pi Foundation). To remotely operate RP-units, we built a WLAN including 16 RP-units and a wireless rooter, WARPSTAR Aterm WR8370N (NEC, Tokyo, Japan) (Fig. 1b). A NAS (LS210DC; Buffalo INC., Nagoya, Japan) was also connected to this WLAN through an Ethernet cable. We accessed and operated the RP-units through Secure Shell (SSH) remote login by using a Windows PC that is connected to this WLAN.

For developing a system that operates external devices, Raspberry Pi 3B, which is equipped with GPIO pins, was used (Fig. 1c). In the present study, we used a green LED lamp, an auto-feeding device (Eheim auto feeder, Eheim, Deizisau, Germany), and an electric circuit including 9 V alkaline battery (6LR61Y(XJ), Panasonic, Kadoma, Japan) as external devices, and we connected some of GPIO-output pins to a custom-made relay unit, which switches on and off external devices by turning on and off the power to the circuit. The block diagram of the relay unit is described in Supplementary Fig. S1. Video-recording was carried out by Raspberry Pi Camera V2.

### Software

**Figure 2:**
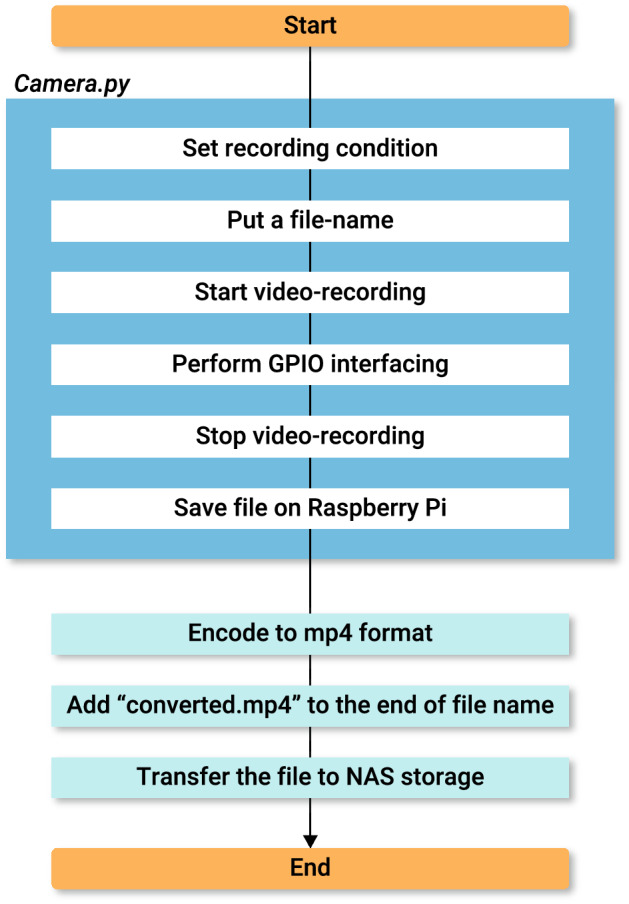
Flowchart of *Record.sh*. Running *Record.sh*, Python script *Camera.py* is run. It executes video-recording, file-naming and GPIO interfacing with the setting in itself. After running *Camera.py*, recorded file in each RP-unit is encoded to mp4 format and transferred to the NAS storage. In this step, the letters “converted.mp4” is added to the end of the file-name for distinguishing files that are completed to be encoded.

All of the shell script for operating Raspberry Pi were Bash Unix shell, and the Python script was written in Python (ver.2.7.13). For video-recording and file-sorting, we created a shell script “*Record.sh*” (Fig. 2). *Record.sh* runs a Python script “*Camera.py*”, which executes video-recording during the recording period and with a camera resolution defined in it. *Camera.py* also defines the file-names and saves the temporary files in a storage of the Raspberry Pi. By default setting, each file-name of contains machine ID, date and time to run *Record.sh*. Machine ID is an assigned number of each RP-unit written in a text-file “*MachineID*”. Furthermore, *Camera.py* includes scripts to operate external devices through GPIO. In this part, the trigger settings for GPIO pins (signal output, the timing and duration of the signal) can be configured. Hence, once *Camera.py* is run, video-recording, file-naming and GPIO interfacing are carried out according to the script. After running *Camera.py*, recorded files in each RP-unit are moved to the NAS storage. Note that conversion of the video file to another format was designed to be done automatically by the main PC in this study. In case of Raspberry Pi 3B, this conversion can be done in the Raspberry Pi itself before moving the video to the NAS. In our experience, Raspberry Pi Zero W takes too much time to achieve format conversion because of its smaller CPU power. We also created a shell script *Setup.sh* for cloning the RP-units easily. When *Setup.sh* is run, interactive command line for setting the machine ID and IP-address appear in the terminal window. After filling them in, *Record.sh* and *MachineID* will be automatically generated. Although a PC keyboard and a display are initially required during this step, they become unnecessary after that, since all operations can be done via SSH remote login (See RP-unit instruction web site (https://github.com/Neuroendo-mdLab/RP-units)).

**Figure 3:**
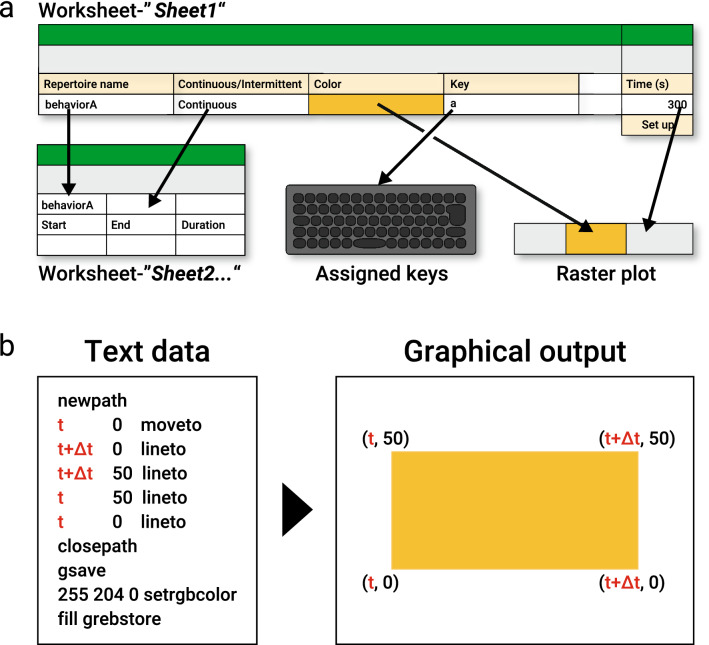
Conceptual diagram of *Ethogramer*. (**a**) Diagram that represents some indices for setting that filled in worksheet- “*Sheet1*” are reflected to each function for behavioral annotation. Data in columns- “Repertoire Name” and “Continuous/Intermittent” in worksheet- “*Sheet1*” configure the setting of columns for annotations of the timing of the behavior in worksheet- “*Sheet2*…”. Key assigning is performed by filling in the letters in column- “Key”. Available keys are shown in Supplementary Information. Color information of the cells in column- “Color” are reflected in drawing raster plots. Filled in Data in cell- “Time (s)” is defined the length of raster plots. After setting these indices, it enabled to perform behavioral annotation by pressing assigned keys. (**b**) Image representing the example of the text-based data exported in EPS format (left, “Text data”) and the graphical image actually outputted when opened by vector graphics editor (right, “Graphical output”). Time information of each behavioral event obtained by pressing keys (for example: a behavioral event is observed from t to t + Δt) are substituted like in the left box, and these text-based data define the coordinates for drawing a square in a raster plot like in the right box.

We made a tool for behavioral annotation by using an Excel macro written in Visual Basic for Applications (VBA), which is here called “*Ethogramer*”. In the worksheet- “*Sheet1*” of the Excel macro, we made four columns and a cell for filling in the following settings: names of behavioral repertoires indicating what we want to analyze (“Repertoire Name”), duration of behavioral repertoire (“Continuous/Intermittent”), Color for drawing raster plot (“Color”), key for recording behavioral repertoire (“Key”), and total time duration of the analysis (“Time (s)”) (Fig. 3a). For the behavioral annotation, we made a user-form (GUI window) “*Analyze*” that was programmed to generate a worksheet (by default, *Sheet 2,3,4*…) for recording the data, based on the settings defined in the worksheet “*Sheet1*”. After pressing “.” to reset the time, the behavioral annotation is carried out by pressing the assigned PC keys based on the worksheet “*Sheet1*”. After completing the behavioral annotation, the raster plot is generated by running another user-form “*Draw_Rasterplot*”. The output for the raster plot is made in Encapsulated Postscript (EPS) format. Since EPS is a text-based format for drawing a graphic, we converted the timing information of each behavioral events to the line widths of the raster plot (Fig. 3b).

### Animals

Two strains of medaka (*Oryzias latipes*), d-rR and Kiyosu were used in the present study. Since d-rR strain has been inbred over 50 years by the researchers, we treated them as a laboratory strain that is influenced by the selection under the artificial environments. In contrast, Kiyosu used in this study was the 5th generation of inbreeding of wild fish that had been caught in Toyohashi City, Aichi Prefecture, Japan, and we treated them as a wild strain. In the both strains, female and male were paired and maintained in fish tanks with water circulation (Labreed, IWAKI Co., Ltd., Tokyo, Japan) under 14-h light/10-h dark photoperiod (light on at 08:00 and off at 22:00) condition at a water temperature of 27 ± 2 °C. These were fed three or four times per day with live brine shrimp and/or commercial flake food (Tetra Medaka-bijin; Spectrum Brands, Yokohama, Japan). All of the fish maintenance and the experiments were conducted in accordance with the protocols approved by the Animal Care and Use Committee of the University of Tokyo (permission number 17-1). The Statement to confirm that all methods were carried out in accordance with relevant guidelines and regulations. Also, the statement to confirm that all methods were carried out in accordance with the ARRIVE guidelines (http://www.nc3rs.org.uk/page.asp?id=1357).

### Analysis of sexual behaviors

**Figure 4:**
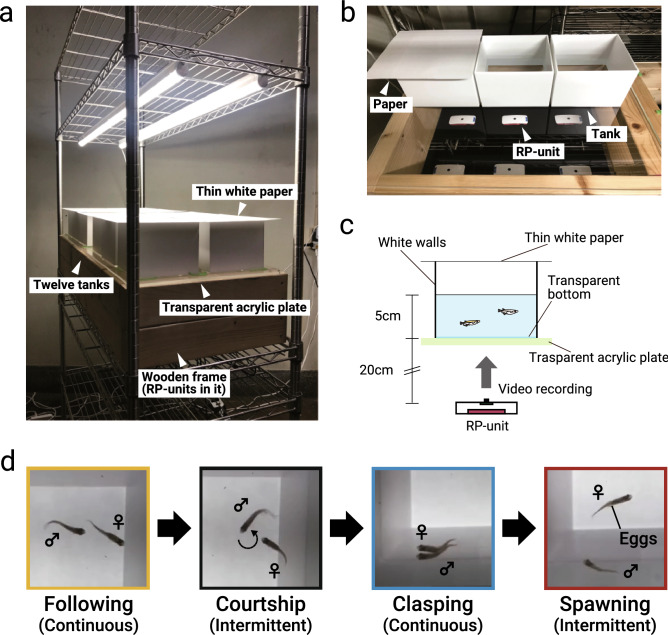
Experimental design for sexual behavior analysis. (**a**) Photograph of the whole experimental equipment for recording the sexual behavior of medaka. Wooden frame that was attached transparent acrylic plate was placed onto the 100 × 45 cm stainless rack. Twelve tanks, with their upper side covered by thin white paper, were put on the acrylic plate, and RP-units were placed inside the wooden frame. (**b**) Photograph of a part of experimental equipment taken from diagonally above. When performing experiments, thin white papers were put onto each tank, like the left tank of this figure. RP-units were placed directly below each tank. (**c**) Schematic illustrating of the experimental equipment. Since both the bottom of the tank and acrylic plate were transparent, it is possible for RP-units to record the videos from ventral side of the medaka. (**d)** Schematic diagram of the sexual behavior sequence of medaka. Images of each of four behavioral repertoires were captured from the video data of one pair of d-rR. Colored frames and words in parentheses are corresponding with the setting of the *Ethogramer*.

The pairs of medaka that showed spawning for three consecutive days before the experimental day were used. On the day before behavioral testing, the pairs were placed and habituated to experimental tanks which had transparent bottom (15 × 15 cm) and white walls (Fig. 4a–c). The water depth was maintained at ~ 5 cm. Each pair of female and male were kept separated until the analysis by putting a transparent perforated partition diagonally set across the tank. These tanks were placed on a transparent acrylic plate, and their upper side was covered with a thin white paper to spread the light evenly from above. The RP-units were put 20 cm under the acrylic plate for each tank to record the behavior from ventral side of medaka. At 09:00 (1.0 h after the onset of the light period) of the following day, we ran *Record.sh* for each RP-unit by SSH remote login. The camera resolution and framerate were set to 640 × 480 pixels and 15 frames per second (fps), respectively in *Camera.py*. The partitions were slowly removed, then the interactions of female and male were recorded for 1 h.

By using *Ethogramer*, we identified the behavioral repertoires and annotated four behavioral repertoires during the sexual behavior; following, courtship, clasping, and spawning (Fig. 4d), while replaying the first 30 min of the videos from the time the partition was removed, with a playing at double speed (actual time required for playing is 15 min per videos). Each behavioral repetoire was identified by referring to the previous studies^22,23^ (also see a brief description of each behavioral repertoire in the “Results” section). We regarded and set following and clasping as continuous behavior, while courtship and spawning as intermittent behavior when using *Ethogramer*. After behavioral annotation for all four behavioral repertoires, we calculated the following behavioral parameters; the latency to first following, the percentage of the time spent for following before spawning, the frequency of courtship before spawning, the percentage of following with successful courtship before spawning, the number of clasping ceased by female before spawning, and the latency to spawning. The parameters were calculated according to the following formulae.

$$\text{Percentage \,of\, time\, spent \,for\, following\, before \,spawning }\left(\text{\%}\right) = \frac{\text{Cumulative time for following before spawning (min)} }{\text{Latency to spawning (min)}} \times 100$$

$$\text{Frequency\, of \,courtship\, before \,spawning }\left({\text{min}}^{-1}\right)= \frac{\text{Number of courtship}}{\text{Latency to spawning (min)}}$$

$$\text{Percentage\, of \,following \,with\, successful\, courtship\, before\, spawning }\left(\text{\%}\right) = \frac{\text{Number of following with courtship in rapid succession } }{\text{Total number of following}} \times 100$$

All of the analyses were performed by double-blind test.

### Statistical analysis

All the values are presented as mean ± standard error of the mean (SEM). Statistical analyses and graph drawing were performed by using R (R Core Team 2020). All of the parameters except for the latency to the first following/spawning were analyzed by Mann–Whitney *U* test and are shown by the whisker and scatter plot. We used the *beeswarm* package (https://cran.r-project.org/web/packages/beeswarm/index.html) to perform the scatter plot. The latency to the first following/spawning was analyzed using Kaplan–Meier plot, and the differences between each Kaplan–Meier curve were tested for statistical significance using Log-Rank test. The *survival* package (https://cran.r-project.org/web/packages/survival/index.html) was used to conduct Kaplan–Meier plots. A *P*-value less than 0.05 was considered statistically significant.

## Results

### Automated behavior recording system with Raspberry Pi enables efficient and reproducible video-recordings

**Figure 5:**
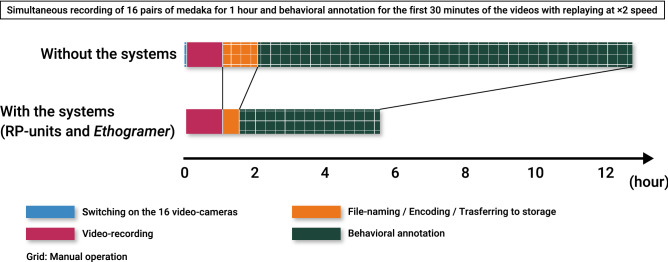
Conceptual diagram of the experimental procedures and their estimated time required for the behavioral analysis with or without the systems. By using the combination of the two systems established in this study, video-recording and their organization were completely automated, and behavioral annotation was drastically shortened and became easier compared to conventional manual behavioral annotation. Without the systems, the observer is forced to manually switch on 16 video cameras. Also, it is necessary to manually perform file-naming/encoding/transferring to strage, which takes approximately 1 h. Furthermore, the behavioral annotation without the *Ethogramer* took 40 min per video. Accordingly, it is expected that behavioral annotation for 16 pairs takes approximately more than ten hours. On the other hand, with the systems, video-recording of 16 pairs of medaka was automatically/simultaneously performed by running *Record.sh* via SSH remote login. Immediately after recording, file-naming, encoding, and transferring to storage were automatically performed, and it took approximately 30 min. Since the behavioral annotation was performed while replaying the first 30 min of each video at double speed by using *Ethogramer*, the time spent for the behavioral annotation of 16 pairs of medaka was only 4 h.

We established an automated behavioral recording system by using Raspberry Pi. Behavioral recording was performed by running *Record.sh* through SSH remote login. In the present study, we simultaneously accessed 16 RP-units and ran *Record.sh* for recording the sexual behavior of medaka (described below in detail). After 1 h video-recording, it only took approximately 30 min to perform required operations automatically for all 16 videos, such as file-naming, encoding and transferring data to the NAS storage (Fig. 5). The 16 video files encoded in the mp4 format were correctly named with the machine ID number and the current timestamp, and stored in the NAS storage. The remote video-recording reduces unintentional human disturbances against the animal behavior, and the automated systematic file-sorting function removes the risk of the erroneous video data storage. In addition, since RP-units are small and easy to install, it requires less space to perform behavioral experiments. In the present study, we made wooden frames that can accommodate twelve tanks and RP-units and carried out simultaneous video-recording of the sexual behavior of twelve medaka pairs in only 100 × 45 cm space (Fig. 4a,b).

### Behavioral annotation tool, *Ethogramer* enables easier and more informative behavioral analysis

We developed *Ethogramer*, which enables behavioral annotation for all behavioral events of interest by only pressing the assigned PC keys while replaying the video. We performed the behavioral annotation of sexual behaviors of 16 pairs of medaka while replaying the first 30 min of the videos played at double speed (Fig. 5). Also, we successfully analyzed the behavioral repertoires that last for certain time, following and clasping, by continuously pressing the assigned key during these two behavioral repertoires. Since we gave the macro the function to record the pressed time and duration of 40 keys (26 alphabet keys (a-to-z), ten numeric keys (0-to-9) and four cross-keys (left/right/up/down)), the observers can assign up to 40 behavioral repertoires and then theoretically annotate up to 40 behavioral repertories during the video replay. Moreover, the raster plots that visualize the behavioral transition along the accurate time scale are generated by only pressing the button. Also, we roughly estimated the time spent for the analysis with or without these systems. In the conventional manual method, video-recording was performed by home-video cameras, and we manually annotated the behavioral events. It was painstaking to annotate the continuous behavior, following and clasping, because we must record both the start and end timing of each behavioral event and calcurate the duration. It took approximately 40 min per 30-min videos, and then it is estimated that it should take more than ten hours for the annotation of all 16 videos. In addition, since it is difficult to generate the raster plot by manual behavioral annotation, it will result in the lack of information to get an overview of the behavioral transition. On the other hand, by using the system established in this study, it is not necessary to switch on the each video cameras, transfer the video data and rename the files. Behavioral annotation took approximately four hours, which is the same as the time for replaying the 16 videos at double speed. Also, the present system allowed us to generate 16 raster plots indicating the behavioral transition of each pair, which makes the behavioral data more informative. Thus, *Ethogramer* enables efficient and informative analysis of behaviors.

### The combination of the Raspberry Pi system and Excel macro enables an efficient and informative comparative analysis of the sexual behavior of medaka between laboratory and wild strains

Here, we show the result of the comparative analysis of the sexual behavior of a teleost medaka as a successful application of the combination of the Raspberry Pi system and Excel macro. Adult medaka is a useful animal model for the studies of reproductive biology, behavioral neuroscience, etc^24,25^. Since the d-rR (domesticated rR) strain of medaka, which is one of the most frequently used laboratory strains, have been empirically recognized to spawn readily compared with the wild strain, and there is an anecdotal report that laboratory strain spawns more readily than the wild ones, we compared the sexual behavior between the two strains, d-rR and Kiyosu as a wild strain.

**Figure 6:**
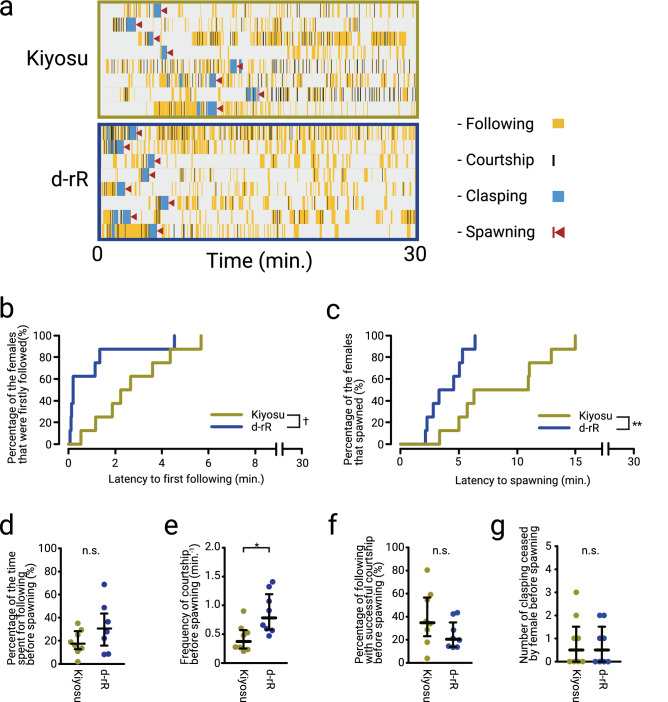
d-rR pairs spawn earlier than Kiyosu pairs, probably because d-rR male is more motivative to mating than Kiyosu male. (**a**) Time-course data of 30-min analyses of sexual behavior are shown as raster plots. Yellow, black, cyan and red bands represent the timing and duration of following, courtship, clasping and spawning, respectively. Red triangles are put on the right of each red bands for easy detection of them. (**b,c**) The latency data were further analyzed using Kaplan–Meier plots. Yellow and Blue curves represent Kiyosu and d-rR, respectively. d-rR pairs showed first following and spawning earlier than Kiyosu pairs (^†^*P* < 0.1, **P < 0.001, by Log-Rank tests). (**d**–**g**) Various parameters were measured and compared between Kiyosu and d-rR. Yellow and Blue dots represent Kiyosu and d-rR, respectively. The frequency of courtship before spawning was significantly higher in d-rR than Kiyosu (**e**), while no significant difference was detected in the percentage of the time spent for following before spawning (**d**), the percentage of following with successful courtship before spawning (**f**), or the number of clasping ceased by female before spawning (**g**) (*P < 0.05, by Mann–Whitney *U* tests).

Male and female medaka pairs show stereotypical sequence of sexual behavior repertoires^22,23^. This sequence begins with the male approaching the female closely from behind and following at the same speed (termed ‘following’), and the male performs courtship display (swimming in front of the female by making a quick circular turn) only when the female slows down swimming (termed ‘courtship’). If the female accepts the male’s courtship, the male grasps the female body by using dorsal and anal fins and vibrates their body to promote spawning (termed ‘clasping’). After the successful clasping, the male and female spawn sperms or eggs respectively, resulting in fertilization (termed ‘spawning’). If the female is not receptive, however, she does not slow down nor accept the male’s courtship but changes direction and rapidly swims away from the male. The female also shows unreceptive behavior in which she ceases clasping before successful spawning. We recorded such behavior repertoires by using RP-units, quantified and analyzed by using *Ethogramer* (Fig. 6). Figure 6a shows the raster plots that indicate the transition of the sexual behavior in the present observation. We found that the latencies to first following and spawning of d-rR were shorter than those of Kiyosu (Fig. 6b,c). It was found that the latency to first following approached significance, suggesting a difference (Kiyosu; 2.763 ± 0.602, d-rR; 0.963 ± 0.540, *P* = 0.070 (Fig. 6B)). Also, significant difference was detected in the latency to spawning (Kiyosu; 8.779 ± 1.492, d-rR; 3.979 ± 0.556, *P* = 0.009 (Fig. 6c)). We further analyzed some indices that reflect male motivation of sexual (Fig. 6d,e), and found that the frequency of courtship before spawning of d-rR is significantly higher than that of Kiyosu (Kiyosu; 0.435 ± 0.085, d-rR; 0.874 ± 0.128, *P* = 0.0148 (Fig. 6e)), while there was no significant difference in the percentage of the time spent for following before spawning (Kiyosu; 18.674 ± 3.80, d-rR; 31.581 ± 7.18, *P* = 0.235 (Fig. 6d)). This result suggests that the d-rR males perform courtship display more frequently than the Kiyosu males. On the other hand, we did not find differences in the indices that reflect female receptivity (Fig. 6f,g). There was no significant difference in the percentage of following with successful courtship before spawning (Kiyosu; 39.221 ± 8.647, d-rR; 24.474 ± 4.376, *P* = 0.195 (Fig. 6f)) and the number of clasping ceased by female before spawning (Kiyosu; 0.875 ± 0.398, d-rR; 0.750 ± 0.313, *P* = 0.957 (Fig. 6g)). These results suggest that the d-rR male is more motivated and tends to get more chances of mating compared to the Kiyosu male, and therefore d-rR pairs spawn more readily than Kiyosu, as shown in Fig. 6c.

## Discussion

In the present study, we established an open-source automated behavioral recording system by using Raspberry Pi. We also used Raspberry Pi for automation of simple experimental treatments such as feeding and illumination by operating external devices through GPIO interfacing. This system drastically reduced experimental operation and enabled us to carry out the efficient and reproducible behavioral recording, which may result in improved quality of behavioral analysis. In addition, we developed the behavioral annotation tool, *Ethogramer*, by using Microsoft Excel macro. It became possible to easily annotate the behavioral events and automatically generate the raster plots, and thus the behavioral analysis became more efficient and informative than the conventional one. We could successfully apply this system to the comparative analysis of the sexual behavior of medaka between laboratory and wild strains and found that the laboratory strain spawn more readily than the wild strain, which is probably caused by the increase in the sexual motivation in males of the laboratory strain.

Since the animals respond to many cues from external environments and change their behavior accordingly, it is essential to minimize human errors caused by experimental operations. Behavioral recording system established in the present study succeeded in automating the greater parts of behavioral experiments including video-recording and in minimizing human errors. Also, the shell script *Record.sh* were programmed to perform not only video-recording but also file-naming, encoding and transferring to the storage, which freed us from such laborious procedures, and the behavioral recordings could be performed more efficiently. In fact, the time spent for video-recording and file-sorting of 16 pairs of medaka was drastically shortened compared to the conventional method. In addition, RP-unit is very small and its smallness also enables us to perform behavioral experiment with less space.

Furthermore, external devices could be controlled by GPIO interfacing with Raspberry Pi, which make it possible to automate various experimental protocols. For the future application, we examined practical ability of the GPIO interfacing. First, we examined the control of external devices via a custom-made relay unit connected to GPIO pins on Raspberry Pi (Supplementary Figure S2). Our system enabled switching on/off the auto-feeding device and green LED lamp as described in “Materials and methods” section (Supplementary Movie 1). Next, to check the time resolution of the relay-switching, we generated the rectangular voltages by changing the timing of switching on the power to the circuit connected to 9 V alkaline battery to switch on/off the relay for various durations (5/10/25/50 ms) and examined the waveform of the rectangular voltages. The rectangular waves with amplitudes of 9 V at 10/20/50/100 Hz were faithfully observed (Supplementary Figure S3). In the present study, we used the relay with 5.0 ms recovery time, so that the result indicated that this relay unit worked as we programmed, without impairing functionality of the relay. Thus, the external devices could be regulated with high time resolution via GPIO interfacing. The system that switches on/off the auto-feeding device and LED lamp can be applied to the learning experiment in which the LED light serves as the signal for feeding synchronized to the timing of switching on the auto-feeding device. While these learning experiments require repetitive conditioning trials, it is possible to automatically execute such trials anytime we want by using “cron”, which is one of the daemons of Raspberry Pi and can be scheduled to run the shell scripts. This option drastically reduce human-tasks in repetitive experiments such as daily observation and repetitive learning. Furthermore, it is also possible to output pseudo-analog signals by using pulse width modulation (PWM) method, which enables us to perform the operations in a manner other than all-or-none. On the other hand, GPIO pins can not only perform signal output but also signal input. Since most of the biological signaling are analog, it is necessary to convert analog to digital signals, which can be accomplished by connecting an analog-to-digital (A/D) converter to GPIO pins on Raspberry Pi. Thus, by customizing Raspberry Pi, it will be possible to develop various experimental systems. In addition, Raspberry Pi can be remotely operated via WLAN, so that it is possible to perform efficient and reproducible analyses by applying the system proposed in the present study. We also created a shell script “*Setup.sh*” for easy setup and cloning RP-units, so that it became easier to start behavioral recording by using RP-units. All the source codes for operating Raspberry Pi are publicly available on GitHub at https://github.com/Neuroendo-mdLab/RP-units.

One of the most important parts of the behavioral analyses is the quantification of the behavior. For this step, there are some full-automated behavioral analysis systems applied for the model animals in which their behavior has been frequently studied, such as rodents^12–16^ or fruit flies^17–21^. Although these systems are useful, these can be applied exclusively for certain behavioral repertoires of specific species and are thus less flexible. On the other hand, we are often faced with experimental needs for analyzing unique/rare behaviors or for using non-model animals. In such cases, we are forced to perform painstaking behavioral annotation that require pausing the video and record the timing and duration manually every time the behaviors occur. However, by using the macro developed in the present study, *Ethogramer,* it is possible to perform behavioral annotation by only pressing the PC keys. It frees us from laborious analyses described above, and the behavioral quantification will be performed much easier. In addition, *Ethogramer* enables us to simultaneously analyze multiple and/or continuous behavioral repertoires, so that such multi-directional perspective makes behavioral analysis more informative. Thus, *Ethogramer* will be especially useful in such situations where the above-mentioned full-automated systems cannot be applied. Moreover, since *Ethogramer* automatically generates raster plots showing the behavioral time course, we can also visually understand the temporal transition of the behavior. Therefore, by using *Ethogramer*, it will be possible to quantify and analyze the behaviors that are difficult to analyze by using conventional analysis methods.

Recently, automated behavioral annotation systems have been established by using machine learning method. For example, *JAABA* is a behavioral annotation tool using a supervised learning method, which can annotate any behaviors that the observer want to analyze^26^. Also, *DeepLabCut* is a sophisticated animal tracking system, which enables us to track the trajectory of animal movement regardless of the animal species^27^. These are very useful tools when the supervised learning is successfully performed. However, these systems require a certain level of knowledge or skills of computing to utilize. In addition, these kinds of machine learning tools often require consistent background and high contrast images for precise tracking. For this reason, it is difficult to apply them to the videos taken in the natural environment, in which many movements other than the objective animal are contaminated. Furthermore, supervised learning systems often require highly repetitive training to perform the appropriate annotation of the behavior that can distinguish subtle differences of animal movement, and the researchers must verify whether the behavioral annotation has been correctly performed by manually checking the video and annotated data. On the other hand, the macro in the present study supports behavioral annotation by human, and it can be applied in any condition as long as human can judge the behaviors. *Ethogramer* is uploaded as Supplementary Information 2. An instruction for *Ethogramer* is described in Supplementary Note 1.

By using the combination of Raspberry Pi and Excel macro in the present study, we analyzed sexual behavior of two strains of medaka. While there is an anecdotal report that laboratory strain spawns more readily than the wild strain, the latency to spawning was significantly shorter in d-rR compared to Kiyosu (Fig. 6c). The latency to first following was shorter almost significantly (Fig. 6b) and the frequency of courtship before spawning were significantly higher in d-rR (Fig. 6e). We analyzed two indices that reflect male motivation of sexual behavior; the percentage of the time spent for following before spawning and the frequency of courtship before spawning, and it was found a significant increase in the frequency of courtship before spawning for d-rR as compared with Kiyosu (Fig. 6d). These data suggest that the d-rR male is more motivated for mating and court more frequently to female compared to Kiyosu male. Medaka is a commonly used teleost fish in various fields of biology, and d-rR is one of the most frequently used laboratory strains of medaka. d-rR has been maintained for decades, and it is well-known among the researchers of medaka that egg collection from d-rR is much easier than that from wild medaka. However, it has been unclear whether d-rR actually spawn more readily than the wild medaka and what underlies this phenomenon. In the present study, the analysis by using the present systems revealed that d-rR male is more motivated for mating compared to the male of wild strain Kiyosu, which are suggested to have afforded more scientifically verifiable hypothesis to the anecdotal reports of the difference in the sexual behavior between laboratory strain and wild individuals. It may be caused by the evolutionary pressure, which is an unconscious selection by researchers for easiness of egg collection. While we did not detect significant difference in the indices that reflect female receptivity in contrast with the indices that reflect male motivation of sexual behavior (Fig. 6f,g), it cannot be sure that there is no difference in female receptivity by only analyzing these two indices in the present study, which were susceptible to male behavior that is different between two strains. To verify this, it is required to analyze these two indices by using F1 hybrid male as mating partner. Since we have been maintaining d-rR and Kiyosu in the completely same breeding method (described in the “Materials and methods” section), the phenomenon found in the present study is unlikely to be caused by epigenetic factors, but rather by genetic factors, and it can be called “domestication.” It is well known that domestication can affect various behaviors of animals^28–32^ including fishes^33,34^. However, to our knowledge, the effect of laboratory domestication on the sexual behavior in fishes have not been clarified yet. Thus, the present study is the first report to suggest a possible effect of domestication on the sexual motivation in a teleost, medaka, and there is no doubt that the behavioral recording/analysis system established here has made a substantial contribution to this finding. It should an important future topic to quantify sexual motivation of multiple laboratory and wild strains to determine whether differences observed in this study is due to domestication effects or not.

In summary, we established automated behavioral recording systems and behavioral annotation tool by using Raspberry Pi and Microsoft Excel macro, respectively. Using the combination of these two, behavioral analysis will be performed more efficiently, which will bring us more informative results and contribute to the progress of various fields of biology.

## Supplementary Information

Supplementary Information 1.

Supplementary Video 1.

Supplementary Information 2.

## Data Availability

All data generated or analyzed during the present study are included in this article and its Supplementary Information files.
