# Supplementary material for: Establishment of open-source semi-automated behavioral analysis system and quantification of the difference of sexual motivation between laboratory and wild strains
Source: Sci Rep. 2021 May 25;11:10894. doi: 10.1038/s41598-021-90225-3 (PMC8149402; doi:10.1038/s41598-021-90225-3)
Supplement: Supplementary file 1 — Supplementary Information 1. [file 41598_2021_90225_MOESM1_ESM.pdf]

Supplementary Information

Title:

Establishment of open-source semi-automated behavioral analysis system and quantification of the difference of sexual motivation between laboratory and wild strains

Authors:

Soma Tomihara[1]*, Yoshitaka Oka[1], Shinji Kanda[2]*

[1]Department of Biological Sciences, Graduate School of Science, The University of Tokyo, 7-3-1 Hongo, Bunkyo, Tokyo 113-0033 Japan

[2]Laboratory of Physiology, Atmosphere and Ocean Research Institute, The University of Tokyo, 5-1-5 Kashiwanoha, Kashiwa, Chiba 277-8564, Japan

Supplementary Method

Automation of feeding and LED lighting interfaced via GPIO on Raspberry Pi

The regulation of auto-feeding devices and LED lamp were performed by using the setup described in the Hardware section. The male of d-rR strain was placed and habituated to the transparent tank (14 × 11 × 7.5 cm) (Supplementary Figures 2a, b). The water depth was maintained at ~8 cm. Raspberry Pi component was located so that the distance between Raspberry Pi Camera V2 and the wall of the tank was 25 cm. The auto-feeding device was placed on the tank, and LED lamp was placed just above the surface of the water. Video-recording and GPIO interfacing were configured in *Camera.py* to switch on auto-feeding device and LED lamp 10 and 15 seconds after *Record.sh* was started (that is, after video-recording was started), respectively. We ran *Record.sh* through SSH remote login. Video-recoding was performed for 30 seconds with the camera resolution of 640 × 480 pixels and 15 fps framerate.

Performance test of a custom-made relay unit

Switching on and off of the power of the circuit using 9 V alkaline battery was performed by using the setup described in the "Hardware" section. We programmed so that rectangular voltages having various wavelengths were instantly generated ten times by consecutive switching of the relay on/off. The wavelength was changed to 10/20/50/100 Hz by editing the script for defining the switching duration of the relay in *Camera.py*. The voltages were measured and converted to the digital data by voltmeter (BNC-2120, National Instruments, Austin, Texas, USA) connected to the circuit. It should be noted that the voltmeter was connected in parallel with the 10kΩ resistor to minimize the current flow to the voltmeter (Supplementary Figure 2c). The waveform analyses of the voltages were performed by using the software for electrophysiology, WinWCP (Dr. John Dempster, University of Strathclyde).

Supplementary Figure 1 Block diagram of the system for operating external devices. The electric current is passed through the relay, accompanied with driving the transistor by the current from GPIO pins (GPIO.Voltage / GPIO.Feeding). At this time, the relay makes a connection with a fixed contact, and the current flows to the entire the circuit. Since current supplied from GPIO is not enough to run the relay, this circuit containing transistor applies current from PW in response to GPIO current flow. GPIO.Voltage, GPIO.LED, GPIO.Feeding indicate the pins that connected to the GPIO pins on Raspberry Pi 3B for operating the electric circuit including the battery, LED lamps and auto-feeding device, respectively. To absorb coil surge to the transistor, diodes are connected between the relay and the transistor.

Supplementary Figure 2 Experimental design for operating external devices by GPIO interfacing. (a) Photograph of the experimental systems for operating external devices. External devices, LED lamp and auto-feeding device were connected to the relay unit via red-and-black leads and switched on/off via GPIO pins and the relay unit. (b) Schematic illustrating of the system of (a). This figure is represented in two dimensions, the width information is omitted. Also, red-and-black leads that connect external devices and the relay unit are omitted for not being complicated. (c) Schematic diagram of the system for testing the performance of relay unit. The rectangular voltages that have various wavelengths were generated by changing the duration of turning on the electricity, and the performance of relay unit was validated. Voltmeter and resistor were connected in parallel in order to decline the current flowed to the voltmeter.

Supplementary Figure 3 Representative waveforms of voltage generated from the circuit that include the battery and was operated via GPIO pins and custom-made relay unit. Left numbers (10/20/50/100 Hz) represent the wavelengths that were set in the Python script, *Camera.py*. Each waveform was reflected the settings for generating the rectangular waves that have specific wavelength.

Supplementary Movie 1 The video represents that the LED lamp and the auto-feeding device were switched on 10 or 15 sec after running *Record.sh*, respectively. The LED lamp was switched on 10 seconds after starting video-recording (0:15), on the other hand, the auto-feeding device was done on 5 seconds after switched on the LED lamp (0:20). These two devices were worked as the setting in *Camera.py* (See also: Materials and Methods section).

Supplementary Figure 1

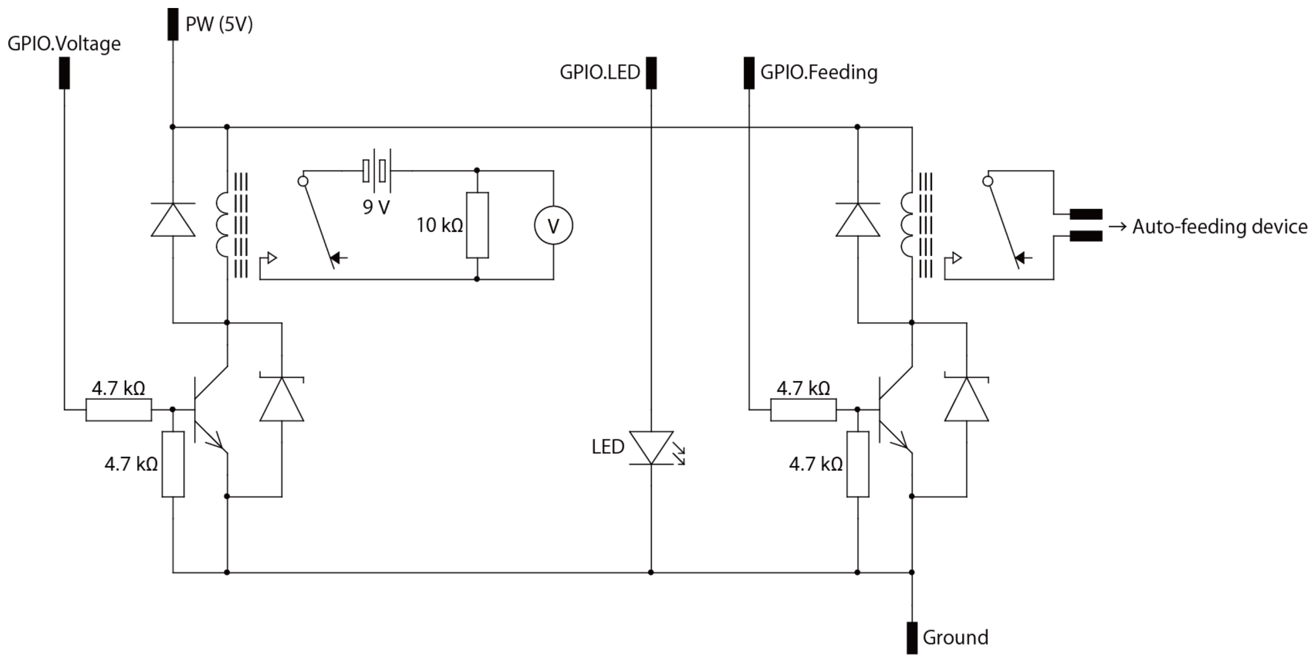

Supplementary Figure 2

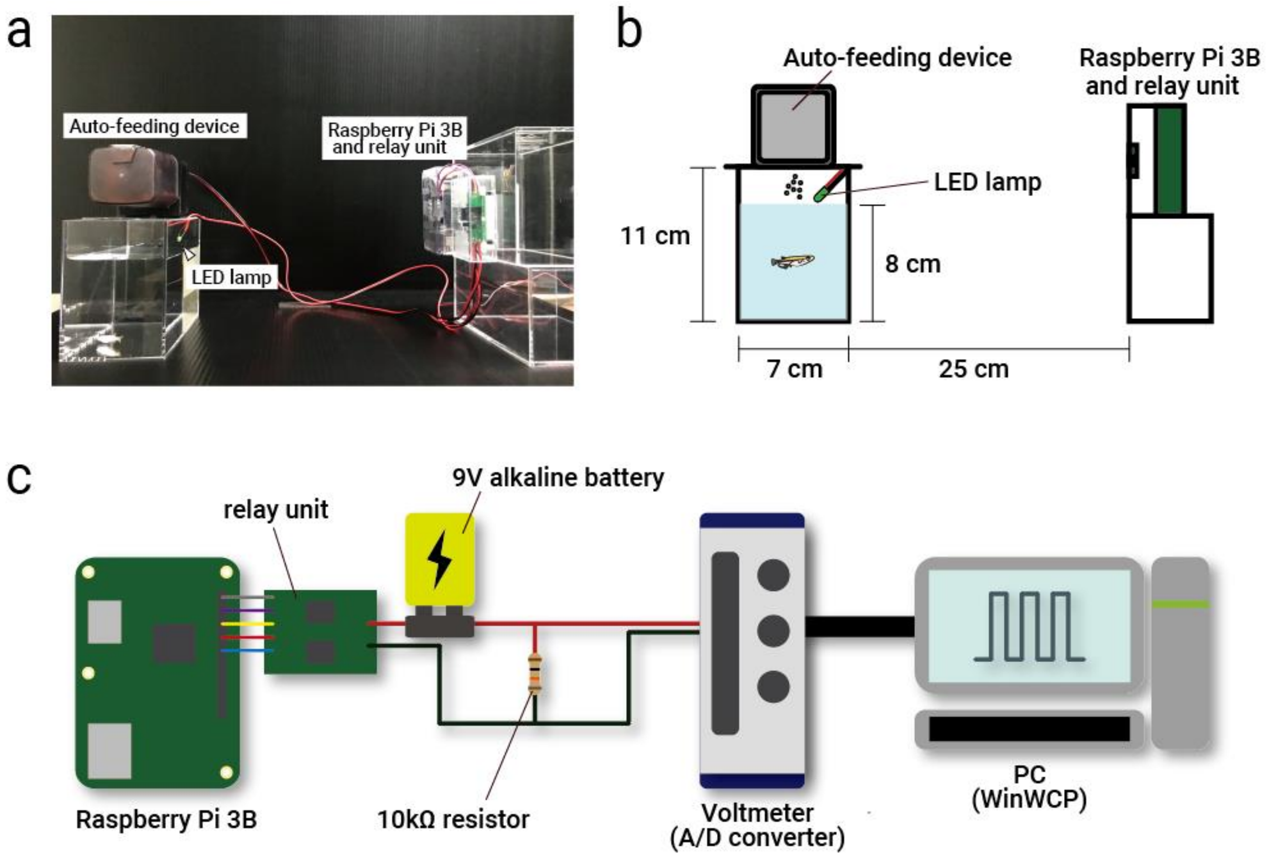

Supplementary Figure 3

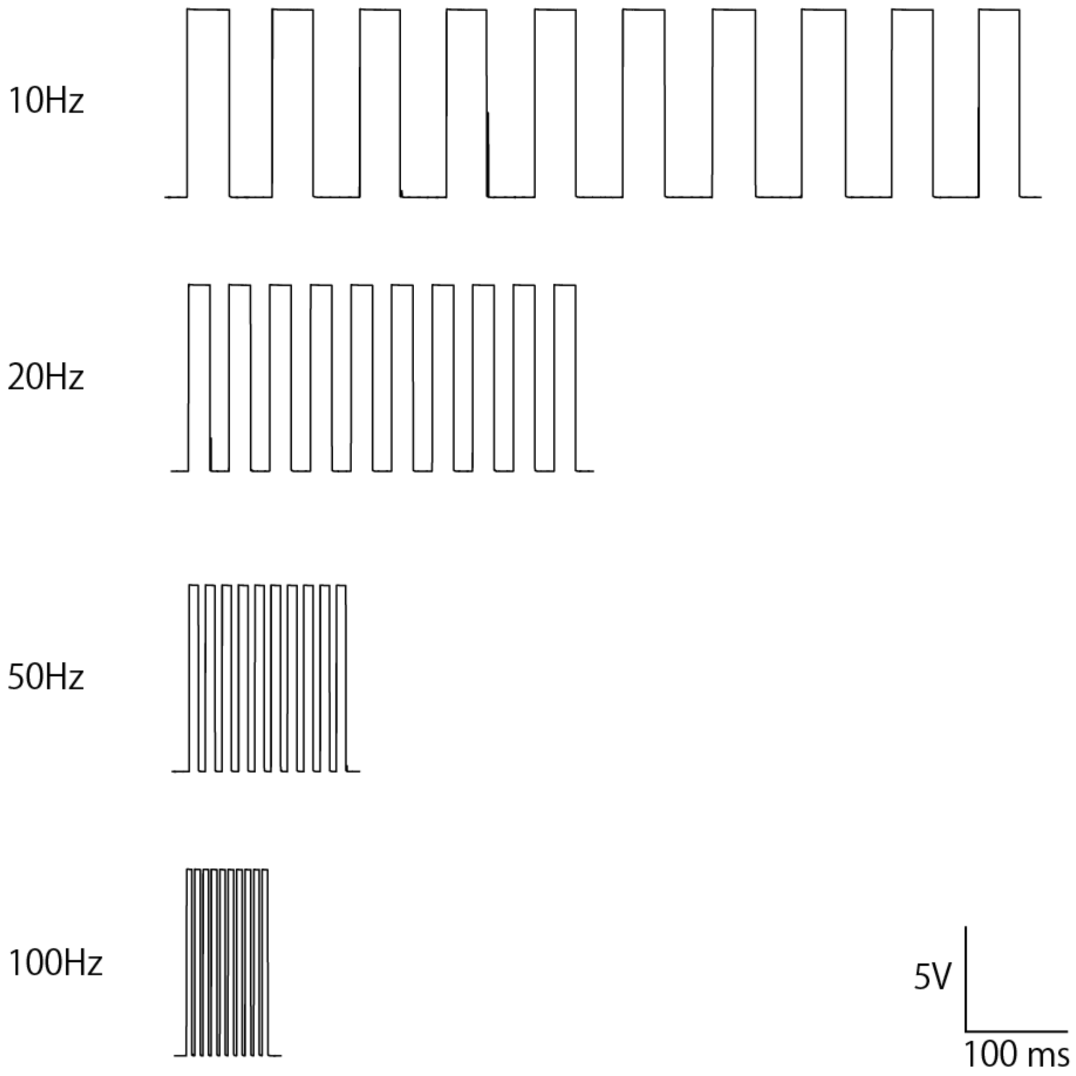

Supplementary Note How to use *Ethogramer*

Before using

- This macro runs on Microsoft Excel.
- Install the Microsoft (MS) Excel. We had confirmed that this macro runs on version.2010 or later.
- Enable macros in Excel.
- The raster plots are output in the Encapsulated PostScript (EPS) format. Prepare the software that support to open EPS files (e.g. Adobe Illustrator, Affinity Designer).

How to use

1. Fill in the name of behavioral repertoires that indicate what you want to analyze in the Column A (Repertoires name).

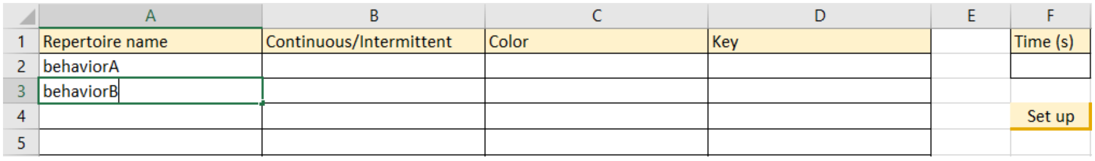

	A	B	C	D	E	F
1	Repertoire name	Continuous/Intermittent	Color	Key		Time (s)
2	behaviorA					
3	behaviorB					
4						Set up
5						

2. Select either "Continuous" or "Intermittent" according to the repertoires in the pull-down menu in the Column B (Continuous/Intermittent). If the behavior lasts for a certain time, select "Continuous." In contrast, select "Intermittent" in case the behavioral repertoire is recorded as instantaneous event. If you choose "Continuous," macro records the time during you are pressing a key.

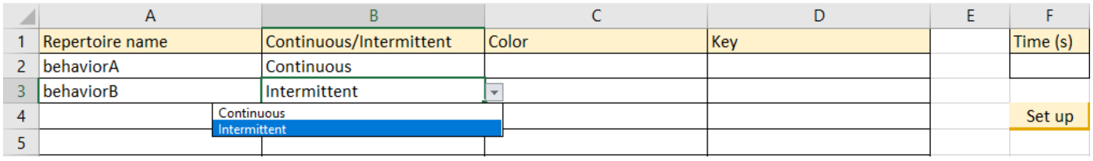

	A	B	C	D	E	F
1	Repertoire name	Continuous/Intermittent	Color	Key		Time (s)
2	behaviorA	Continuous				
3	behaviorB	Intermittent				
4						Set up
5						

3. Assign color for drawing raster plots by changing the color of cell in the Column C (Color). The theme color of raster plots is set to gray (#E6E6E6) by default and we do not recommend to select colors close to gray.

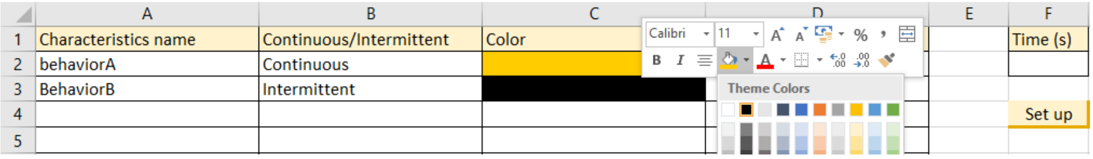

	A	B	C	D	E	F
1	Characteristics name	Continuous/Intermittent	Color			Time (s)
2	behaviorA	Continuous				
3	BehaviorB	Intermittent				
4						Set up
5						

4. Register the keyboard's key to each behavioral repertoire in the Column D (Key). You can use 26 alphabet keys (A-Z), 10 numeric keys (0~9) and 4 cross-key (left/right/up/down). See the Table.1 (at the end of this document) for how to assign keys.

	A	B	C	D	E	F
1	Repertoire name	Continuous/Intermittent	Color	Key		Time (s)
2	behaviorA	Continuous		a		
3	behaviorB	Intermittent		b		
4						Set up
5						

5. Fill in the time for analysis. This information is used for setting of the length of raster plots.

	A	B	C	D	E	F
1	Repertoire name	Continuous/Intermittent	Color	Key		Time (s)
2	behaviorA	Continuous		a		300
3	behaviorB	Intermittent		b		
4						Set up
5						

6. Double-click the "Set up" cell. Another worksheet and a pop-up ("*Analyze*") window for analysis will appear.

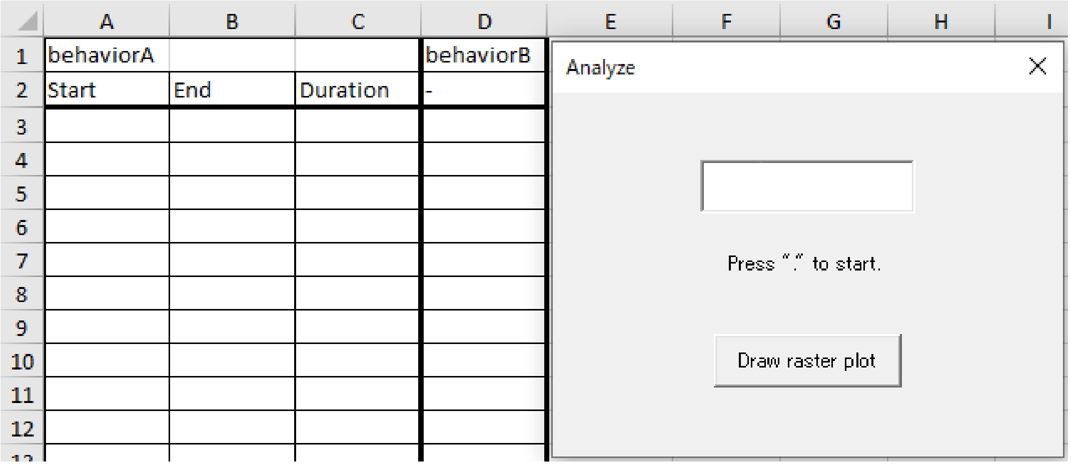

7. Start analysis by pressing period (".")-key. Watch the movies and press the registered keys when each behavioral repertoire is shown. When the continuous behavior is observed, keep pressing the key while observing.

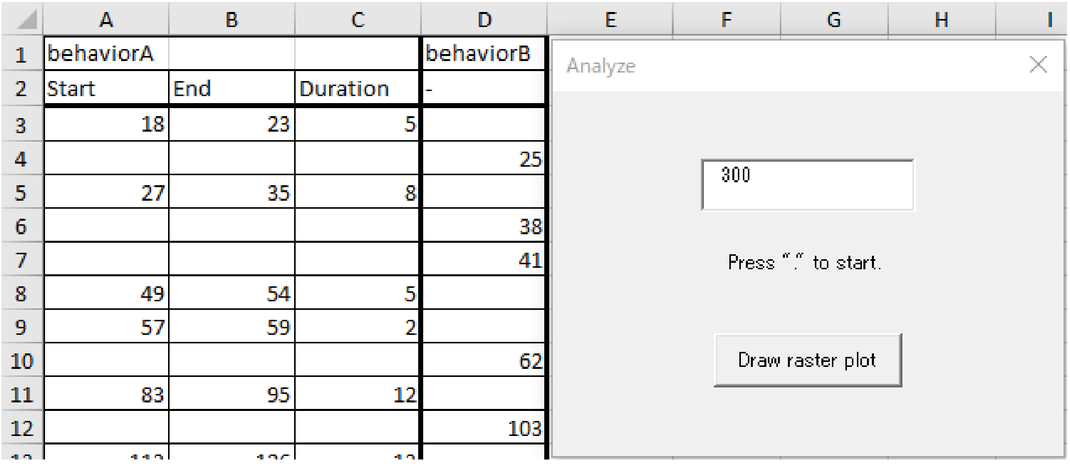

8. Click the “Draw raster plot” button in the *Analyze* window. Another pop-up window (”*Draw_rasterplots*”) for generating raster plots appears. Then, click the “Save” button to generate raster plot data in the large box below and dialog box for file-saving appears. Note that these generated texts are the content of raster plot EPS file.

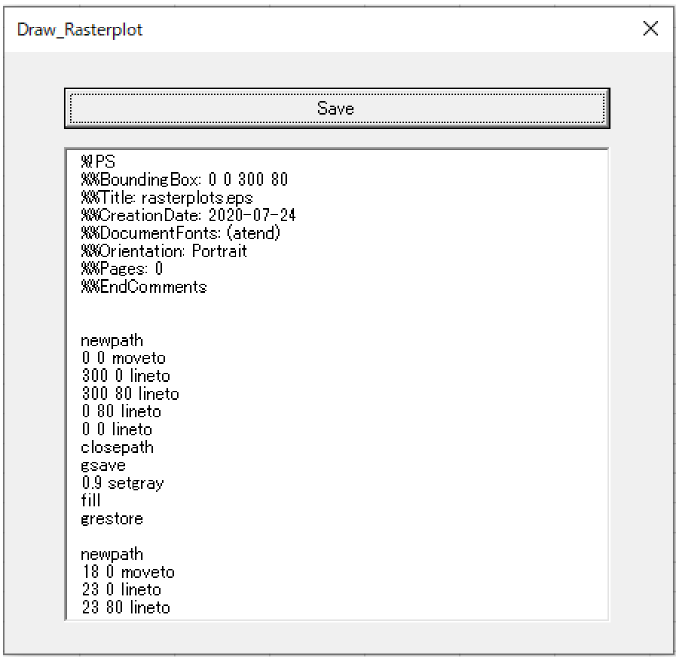

9. Open the EPS file with a vector graphics software and edit.

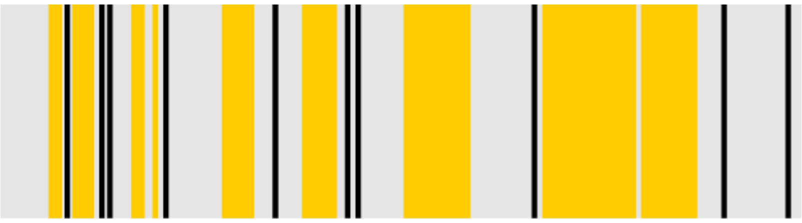

Alphabet keys		Numeric keys		Cross keys	
Assigned key	Filled in letter	Assigned key	Filled in letter	Assigned key	Filled in letter
A key	a	0 key	N0	↼ key	LEFT
B key	b	1 key	N1	↾ key	UP
C key	c	2 key	N2	⇀ key	RIGHT
D key	d	3 key	N3	⇂ key	DOWN
E key	e	4 key	N4		
F key	f	5 key	N5		
G key	g	6 key	N6		
H key	h	7 key	N7		
I key	i	8 key	N8		
J key	j	9 key	N9		
K key	k				
L key	l				
M key	m				
N key	n				
O key	o				
P key	p				
Q key	q				
R key	r				
S key	s				
T key	t				
U key	u				
V key	v				
W key	w				
X key	x				
Y key	y				
Z key	z				

Table.1 Key assignment
